# Role of Lymphopenia in Early prediction of Infection Following Orthotopic Liver Transplantation in Cirrhotic Patients

**DOI:** 10.3389/ti.2025.14372

**Published:** 2025-05-12

**Authors:** Mikhael Giabicani, Clara Timsit, Léa Copelovici, Pauline Devauchelle, Marion Guillouët, Marina Hachouf, Sylvie Janny, Juliette Kavafyan, Stéphanie Sigaut, Tristan Thibault-Sogorb, Safi Dokmak, Federica Dondero, Mickael Lesurtel, Olivier Roux, François Durand, Emmanuel Weiss

**Affiliations:** ^1^ Department of Anaesthesiology and Critical Care, Beaujon Hospital, DMU Parabol, AP-HP Nord, and Université Paris Cité, Paris, France; ^2^ Centre de Recherche des Cordeliers, Sorbonne Université, Université, Paris Cité, Inserm, Laboratoire ETREs, Paris, France; ^3^ Departement of HPB Surgery & Liver Transplantation, AP-HP, Beaujon Hospital, DMU DIGEST, Université Paris-Cité, Clichy, France; ^4^ Université Paris-Cité, Inserm, Centre de Recherche sur l’Inflammation, UMR 1149, Paris, France; ^5^ Service d’Hépatologie, AP-HP, Hôpital Beaujon, DMU DIGEST, Centre de Référence des Maladies Vasculaires du Foie, FILFOIE, ERN RARE-LIVER, Clichy, France

**Keywords:** infection, lymphopenia, cirrhosis associated immune dysfunction, orthotopic liver transplantation (OLT), cirrhosis

## Abstract

Infections remain a main cause of morbidity and mortality following orthotopic liver transplantation (OLT). Patients with end-stage liver cirrhosis exhibit a deregulation of their immune response, making them more susceptible to infections. From a prospective database, we retrospectively assessed the ability of preoperative lymphopenia, as a marker of this immune dysregulation, to predict the occurrence of early postoperative bacterial infections during post-OLT ICU hospitalization in patients with cirrhosis. Between January 2011 and December 2021, we included 445 patients. Post-OLT infections occurred in 92 patients (21%) and were mainly represented by bacteriemia (39%), pneumonia (37%) and surgical site infection (30%). Preoperative lymphocyte count ≤1.150 × 10^9^/L was identified as an independent risk factor, as well as preoperative encephalopathy, intraoperative RBC transfusion >2 and intraoperative maximum norepinephrine dose >0.5  μg.kg^−1^.min^−1^ (all p < 0.05). Bootstrap analysis validated these results (p < 0.05). The risk factors were integrated into the PRELINFO score which was associated with the risk of infection (p < 0.05). The depth of preoperative lymphopenia was also associated with the risk of infection and postoperative correction of lymphopenia was slower in patients who developed an infection than in those who did not. Preoperative blood lymphocyte count should be incorporated into the assessment of the risk of early post-OLT bacterial infections.

## Graphical Abstract

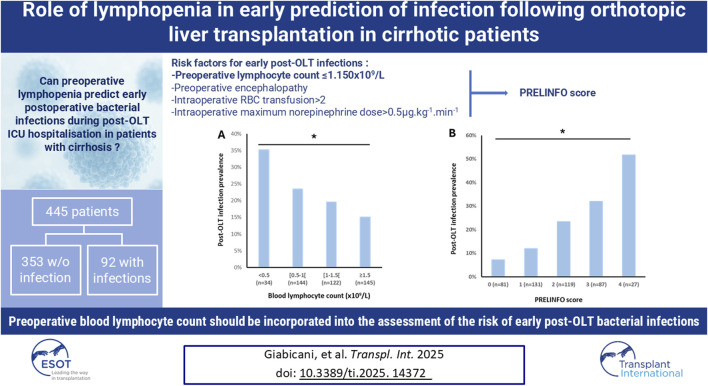

## Introduction

The occurrence of infection following orthotopic liver transplantation (OLT) remains one of the main postoperative complications affecting patient morbidity and mortality [1, 2]. The incidence of infectious complication after OLT reported in literature ranges from 20% to 70% [3–5] and in more than 2/3 of cases, these infections are of bacterial origin [6, 7]. Especially when they occur during intensive care hospitalization, infections following OLT increase the risk of early death, the duration under mechanical ventilation and the intensive care unit (ICU) length of stay [3, 4]. Then, in an era where the trend is towards personalized medicine and “fast-tracking” strategy bypassing a systematic ICU hospitalization after OLT [8–10], early identification of patients at risk to develop infection after OLT would be useful to tailor their perioperative management and their immunosuppressive regimen.

Different risk factors for bacterial infections after OLT have been proposed in the literature including poor clinical conditions of the recipient (high Model for End-Stage Liver Disease (MELD) score, Acute-on-Chronic Liver Failure (ACLF), sarcopenia…), the complexity of the surgical procedure (blood transfusion, cold ischemia time, duration of surgery, type of biliary anastomosis…), and postoperative risk factors (type of immunosuppression therapy, ICU length of stay, biliary complication…) [11]. The recipient’s immune system could also play a particularly important role [12]. Patients with end-stage liver cirrhosis, which is the main indication for OLT in Europe [13], are known to exhibit a deregulation of their immune response described under the term “cirrhosis associated immune dysfunction” (CAID) [14, 15]. One of the consequences of CAID is lymphopenia, which has led to the absolute blood lymphocyte count being considered one of the simplest surrogate markers for assessing CAID [16–18]. However, the impact of absolute lymphopenia on the early onset of bacterial infections after OLT has been poorly studied.

The aim of this study was to analyze the ability of preoperative blood lymphocyte count to predict the occurrence of early postoperative bacterial infections after OLT in patients with cirrhosis.

## Patients and Methods

### Study Design and Patients

We performed a retrospective monocenter (Beaujon Hospital, Clichy, France) observational study from a prospective database from January 2011 to December 2021. This study was conducted in accordance with both the Declarations of Helsinki and Istanbul and was approved by the local ethics committee, which waived the need for written informed consent (Institutional Review Board—IRB 00006477—of HUPNVS, Paris 7 University, AP-HP— 13-020).

All patients older than 18 years who received an OLT for underlying cirrhosis were included. The non-inclusion criteria were: history of previous liver transplantation, multiple organ transplantation (combined liver-kidney, liver-lung or liver-heart transplantation), primary graft non-function [19], immediate preoperative infection (including ACLF patients with an infectious trigger) or suspicion of intraoperative infection, and unknown preoperative blood lymphocyte count.

### Data Collection

For each patient, clinical and biological data were recorded preoperatively, intraoperatively and postoperatively during ICU stay. Preoperative data included demographic parameters, etiology and severity of underlying liver disease as assessed by MELD score and ACLF before OLT. Intraoperatively, data such as duration of surgery, blood loss and number of packed red blood cell (RBC) units transfused during surgery, cold and warm ischemia times, type of biliary reconstruction (duct-to-duct or Roux-en-Y anastomosis) and reperfusion syndrome were recorded.

Biological data included biochemical, hematological and bacteriological data. Blood cell count data were collected retrospectively from the immediate preoperative period until postoperative day 7 using medical charts.

Patients were followed up during their postoperative ICU stay to record: usual ICU severity score (SAPS II) at admission, postoperative morbidity: infection occurring during the ICU hospitalization and time between OLT and infection, acute renal failure and duration of renal-replacement therapy, mechanical ventilation duration, vasopressor infusion duration and ICU length of stay. Only the first episode of post-OLT infection was considered. Mortality was assessed at day 30 and day 90 after OLT.

### Definitions

Pretransplant lymphopenia was defined as a preoperative blood lymphocyte count <1.50 × 10^9^/L.

The criteria used to define ACLF were those published by Moreau et al. [20].

All bacterial infections occurring during ICU hospitalization were investigated. Importantly, all infections were diagnosed on the basis of a clinical suspicion that was confirmed by the isolation of a bacteria from microbiological culture. We have used the same definitions in our work as those published in a previous study [21].

The diagnosis of pneumonia was based on Infectious Disease Society of America guidelines [22]. It was consistently suspected on clinical criteria (2 or more of the following characteristics: temperature >38.3°C or <36°C, leukocyte count >10 G/L or <4 G/L, and purulent respiratory secretions) and radiological findings (new lung infiltrate on chest radiography). It was confirmed by a lower respiratory tract microbiological sample (blind protected bronchial sampling (BPSS) or bronchoalveolar lavage (BAL)). The diagnostic thresholds for BPSS and BAL quantitative cultures were 10^3^ Colony-Forming Units (CFU)/mL and 10^4^ CFU/mL respectively.

Surgical-site infections (SSI) were defined according to the CDC National Nosocomial Infections Surveillance criteria [23] as superficial, deep or organ/space. In this study, only deep and organ/space SSI were considered. Their diagnosis was made on the basis of clinical (at least 1 of the following signs or symptoms: fever >38°C, localized pain or tenderness) and biological (leukocytosis, liver exams abnormalities) signs. It was confirmed by the isolation of bacteria from biliary fluid, from peritoneal fluid containing >250 polymorphonuclear cells/mm^3^, or from an intra-abdominal abscess or collection. All these microbiological samples were obtained aseptically when surgical or radiological drainages were performed, or by percutaneous aspiration. No culture of fluid obtained through a previous drain was considered.

Urinary tract infections (UTI) were diagnosed based on the guidelines of the Infectious Disease Society of America [24]. Of note, asymptomatic bacteriuria and uncomplicated UTI were not considered in this work. Complicated UTI were defined by the presence of signs and symptoms compatible (new onset or worsening of fever, rigors, altered mental status, malaise or lethargy, flank pain, costo-vertebral angle tenderness, hemodynamic instability, leukocytosis) with no other source of infection along with a significant growth of a uropathogen (≥10^3^ CFU/mL). Catheter-related UTI diagnosis was requiring signs and symptoms in presence of indwelling urinary catheters and presence of ≥10^3^ CFU/mL in a single catheter urine specimen or in a midstream urine in case of urinary catheter removal in the previous 48 h.

Finally, bacteremia was defined as a positive peripheral blood culture bottle result together with clinical and biological signs of infection.

### Antimicrobial Prophylaxis Protocol

All patients received an intraoperative antimicrobial prophylaxis. According to our local protocol and following the results of our previous studies [21, 25], the patients received either cefoxitin or a targeted antimicrobial prophylaxis tailored to cover ESBL-E (Extended-Spectrum β-Lactamase-producing *Enterobacteriaceae*) colonizing bacteria in case of known preoperative rectal carriage (carbapenems, piperacillin-tazobactam or cefoxitin according to the antibiotic susceptibility testing). The duration of prophylaxis was limited exclusively to the intraoperative period for all patients.

### Immunosuppressive Regimen

For all patients, immunosuppressive regimen consisted in a triple therapy combining glucocorticoids, mycophenolate mofetil and either tacrolimus (in case of normal renal function) or basiliximab (in case of acute or chronic renal failure). All patients received an intravenous bolus of 5 mg.kg^−1^ glucocorticoids intraoperatively and on admission to the ICU, followed by a daily dose reduction. At D7, all patients received only 20 mg glucocorticoids. Mycophenolate mofetil was administered enterally at a dose of 1,500 mg twice daily, or 1,000 mg twice daily intravenously when enteral administration was not possible. For patients receiving basiliximab, a 20 mg dose was administered intravenously on D0 and D4. For these patients, tacrolimus was introduced no later than D7. For patients receiving tacrolimus initially, treatment was initiated at a daily dose of 0.025 mg.kg^−1^ administered by enteral route. Dosage was adjusted daily according to residual tacrolimus blood levels. The target residual tacrolimus level was individually adjusted according to the patient’s hematocrit and protidemia (ranging, in extreme cases, from 3 to 11 μg/L).

### Outcome Variables

The primary outcome was the occurrence of a bacterial infection during the ICU hospitalization following OLT. Secondary outcomes were mechanical ventilation duration, vasopressor infusion duration, ICU length of stay, need for and duration of renal-replacement therapy and ICU mortality within 30 days and 90 days.

### Statistical Analyzes

Data were compared using Mann-Whitney U test or Kruskal-Wallis test for continuous variables and using Fisher’s exact test for qualitative variables. Variables achieving a *p* value <0.05 in univariate analysis were introduced into a multivariable logistic regression model with backward elimination (exit *p* = 0.05) in complete cases. Potential collinearity between variables was checked, and the more clinically relevant variable was retained in the case of collinearity. Significant continuous variables identified in the univariable analysis were dichotomized to optimize their sensitivity and specificity using the Youden index with the creation of ROC curves. Then, variables with *p* values <0.05 by multivariable logistic regression were included in an infection risk score, using the beta coefficient to build the score. A bootstrap analysis with 2000 resampling was used to confirm the result of the multivariable logistic regression model.

Results are expressed as number and percentage or median and interquartile range. All tests were 2-sided and used a significance level of 0.05. Data handling and analysis were performed with SPSS 22.0 (SPSS Inc., Chicago, IL).

## Results

### Patients’ Characteristics

**FIGURE 1:**
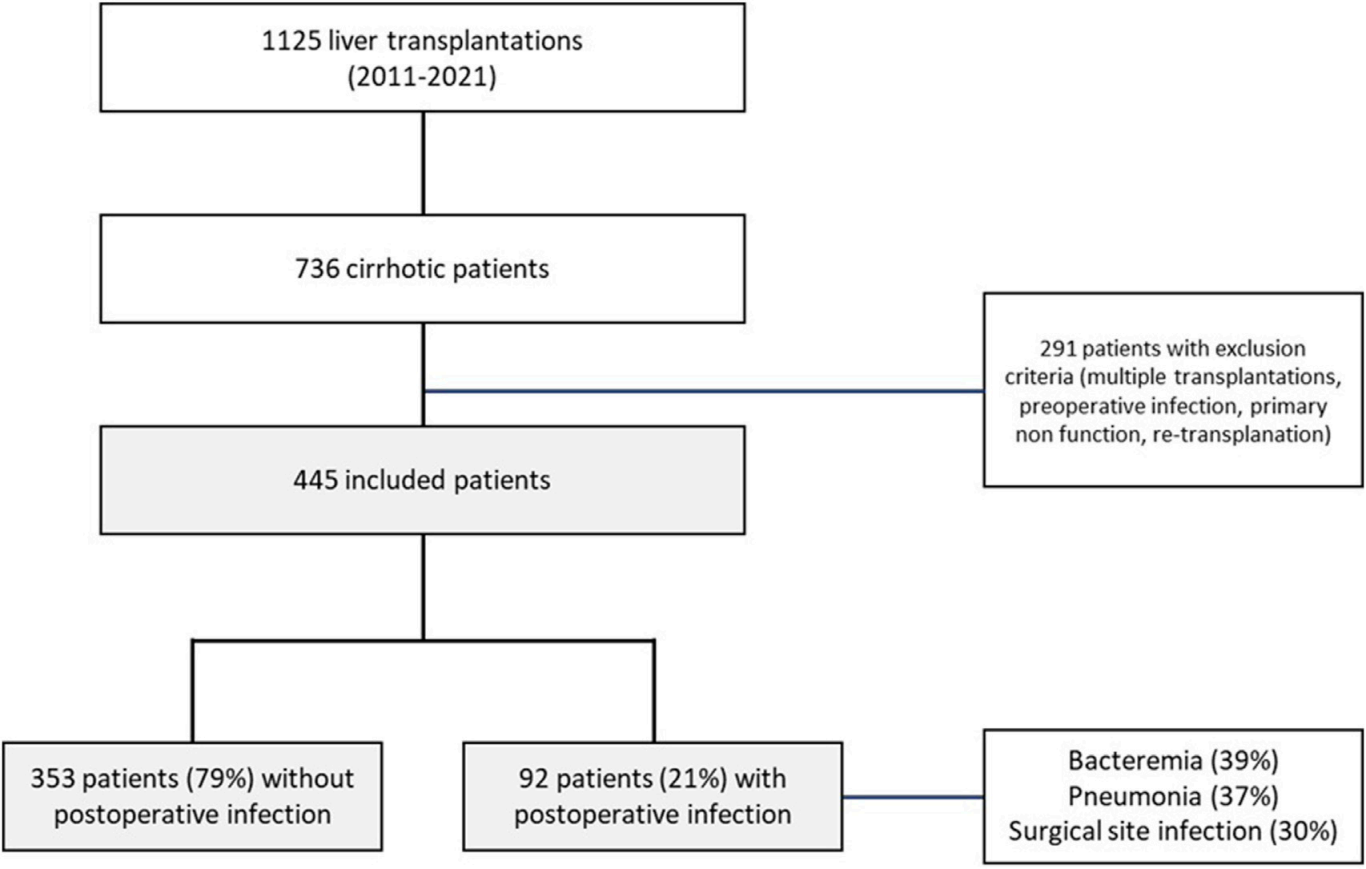
Flow chart.

Of the 1,125 patients transplanted at Beaujon Hospital during the study period, 736 had underlying cirrhosis. After exclusion of the 291 patients who did not meet the inclusion criteria, 445 patients were finally included in the study (Figure 1). Population characteristics are displayed in Table 1. The main cause of liver disease was alcohol-related cirrhosis (55%) and median MELD score on the day of transplantation was 14 [10–20]. Thirty-five (8%) patients underwent liver transplantation for ACLF. Donor information is presented in Supplementary Table S1.

**TABLE 1:** Patients’ main characteristics and univariate analysis.

Characteristics	All (n = 445)	Post-LT infection (n = 92)	Absence of post-LT infection (n = 353)	*p*
Baseline characteristics				
Age (years)	57 [51–63]	58 [50–63]	57 [52–63]	0.897
Male sex, n (%)	343 (77)	70 (76)	273 (77)	0.799
BMI (kg.m^−2^)	26 [24–30]	27 [24–31]	26 [24–30]	0.425
Malnutrition, n (%)	164 (37)	42 (47)	122 (35)	**0.050**
Diabetes, n (%)	105 (24)	23 (25)	82 (23)	0.736
HIV coinfection, n (%)	8 (2)	2 (2)	6 (2)	0.763
COPD, n (%)	8 (2)	2 (2)	6 (2)	0.767
Cause of cirrhosis, n (%) • Excessive alcohol consumption • Metabolic syndrome • HCV infection • HBV infection • Auto-immune hepatitis • Cholestatic liver disease	243 (55) 115 (26) 119 (27) 61 (14) 19 (4) 15 (3)	55 (60) 27 (29) 20 (21) 7 (8) 5 (5) 3 (3)	188 (53) 88 (25) 99 (28) 54 (15) 14 (4) 12 (3)	0.263 0.389 0.224 0.056 0.535 0.948
HCC, n (%) • HCC compensated cirrhosis	222 (50) 133 (30)	36 (39) 19 (21)	186 (53) 114 (32)	**0.021** **0.030**
Preoperative ascites, n (%)	140 (31)	33 (36)	107 (30)	0.307
Preoperative encephalopathy, n (%)	169 (38)	49 (53)	120 (34)	**<0.001**
Decompensated cirrhosis, n (%)	312 (70)	73 (79)	239 (68)	**0.030**
History of SBP, n (%)	76 (17)	22 (24)	54 (15)	**0.050**
Severity of cirrhosis • MELD • MELD≥ 25, n (%) • ACLF, n (%)	14 [10–20] 57 (13) 35 (8)	17 [11–23] 21 (23) 15 (16)	13 [10–19] 36 (10) 20 (6)	**0.001** **<0.001** **<0.001**
Pre-LT blood count				
Hemoglobin (g/L) Hemoglobin<11 g/L, n (%)	119 [101–138] 149 (33)	112 [96–132] 44 (48)	121 [103–138] 105 (30)	**0.011** **<0.001**
Platelets (x10^9^/L)	91 [67–130]	81 [61–127]	95 [69–132]	0.077
Leucocytes (x10^9^/L)	5.20 [4.00–6.80]	5.15 [3.80–7.08]	5.20 [4.05–6.80]	0.966
Neutrophils (x10^9^/L)	3.10 [2.29–4.20]	3.15 [2.42–4.45]	3.10 [2.20–4.10]	0.333
Lymphocytes (x10^9^/L)	1.13 [0.74–1.60]	0.98 [0.60–1.47]	1.20 [0.78–1.66]	**0.006**
Lymphocytes≤1.15 × 10^9^/L, n (%)	231 (52)	61 (66)	170 (48)	**0.002**
Monocytes (x10^9^/L)	0.57 [0.40–0.80]	0.59 [0.37–0.80]	0.57 [0.40–0.79]	0.635
Intraoperative characteristics				
Surgery duration (min)	315 [270–360]	320 [270–387]	310 [274–360]	0.375
Cold ischemia time (min)	425 [357–536]	432 [374–540]	420 [351–535]	0.570
Warm ischemia time (min)	45 [38–53]	45 [36–55]	45 [39–52]	0.904
Blood loss (mL) Blood loss≥750	1,000 [500–1,500] 254 (57)	1,000 [788–2000] 63 (68)	900 [500–1,400] 191 (54)	**<0.001** **<0.001**
RBC transfusion, n (%)	223 (50)	60 (65)	163 (46)	**<0.001**
Number of RBCs units transfused (U)	1 [0–2]	2 [0–4]	0 [0–2]	**<0.001**
RBCs transfusion>2U (%)	99 (22)	36 (39)	63 (18)	**<0.001**
Reperfusion syndrome, n (%)	224 (50)	50 (54)	174 (49)	0.348
Maximum norepinephrine dose (µg.kg^−1^.min^−1^) Maximum norepinephrine dose>0.5 μg.kg^−1^.min^−1^, n (%)	0.55 [0.29–0.93] 239 (54)	0.71 [0.48–1.19] 66 (72)	0.50 [0.25–0.85] 173 (49)	**<0.001** **<0.001**
Biliary reconstruction, n (%) • Duct-to-duct • Roux-en-Y anastomosis	408 (92) 6 (1)	86 (93) 1 (1)	322 (91) 5 (1)	0.792 0.792

Mann-Whitney U test used for continuous variables. Chi-square test used for categorical variables. Results are expressed as number (percentage) or median [interquartile range]. p-value <0.05 was considered significant. p-values in bold are significant. BMI, body mass index; HIV, Human Immunodeficiency Virus; COPD, chronic obstructive pulmonary disease; HCV, hepatitis C virus; HBV, hepatitis B virus; HCC, hepatocellular carcinoma; SBP, spontaneous bacterial peritonitis; MELD, Model for End-stage Liver Disease; ACLF, Acute on Chronic Liver Failure; RBC, red blood cells.

Post-OLT infection occurred in 92 patients (21%) during ICU hospitalization, including 9 patients who developed septic shock. These infections were mainly represented by bacteriemia (39%), pneumonia (37%), surgical site infection (30%) and UTI (26%). Species involved in post-OLT infections were mainly *Enterobacterales* and *Enterococci*. The median time between OLT and infection was 5 [4–7] days. Two patients developed a fungal infection in addition to the bacterial infection, and no patient developed a viral infection. There was no difference in the occurrence of post-OLT infection between patients receiving basiliximab (31%) and those receiving tacrolimus (69%) as an immunosuppression induction regimen: 24% and 19% respectively (*p* = 0.163).

Complications and mortality are presented in Supplementary Table S2. Patients who developed post-OLT infection had higher 30-day and 90-day mortality rates than those who did not. Moreover, durations of mechanical ventilation, vasopressor infusion and ICU stay were longer, and renal-replacement therapy requirement was more frequent among patients who developed an infection. Fifteen patients (3%) had died by postoperative day 90. Among them, 13 patients had died during ICU-hospitalization.

### Association Between Preoperative Blood Lymphocyte Count and Prevalence of Post-OLT Infections

Univariate analysis investigating factors associated with post-OLT infections is displayed in Table 1. Patients who developed a post-OLT infection had a lower preoperative blood lymphocyte count than those who did not. Patients with a blood lymphocyte count of less than 1.150 × 10^9^/L had an almost 2-fold increased risk of post-operative infection.

**FIGURE 2:**
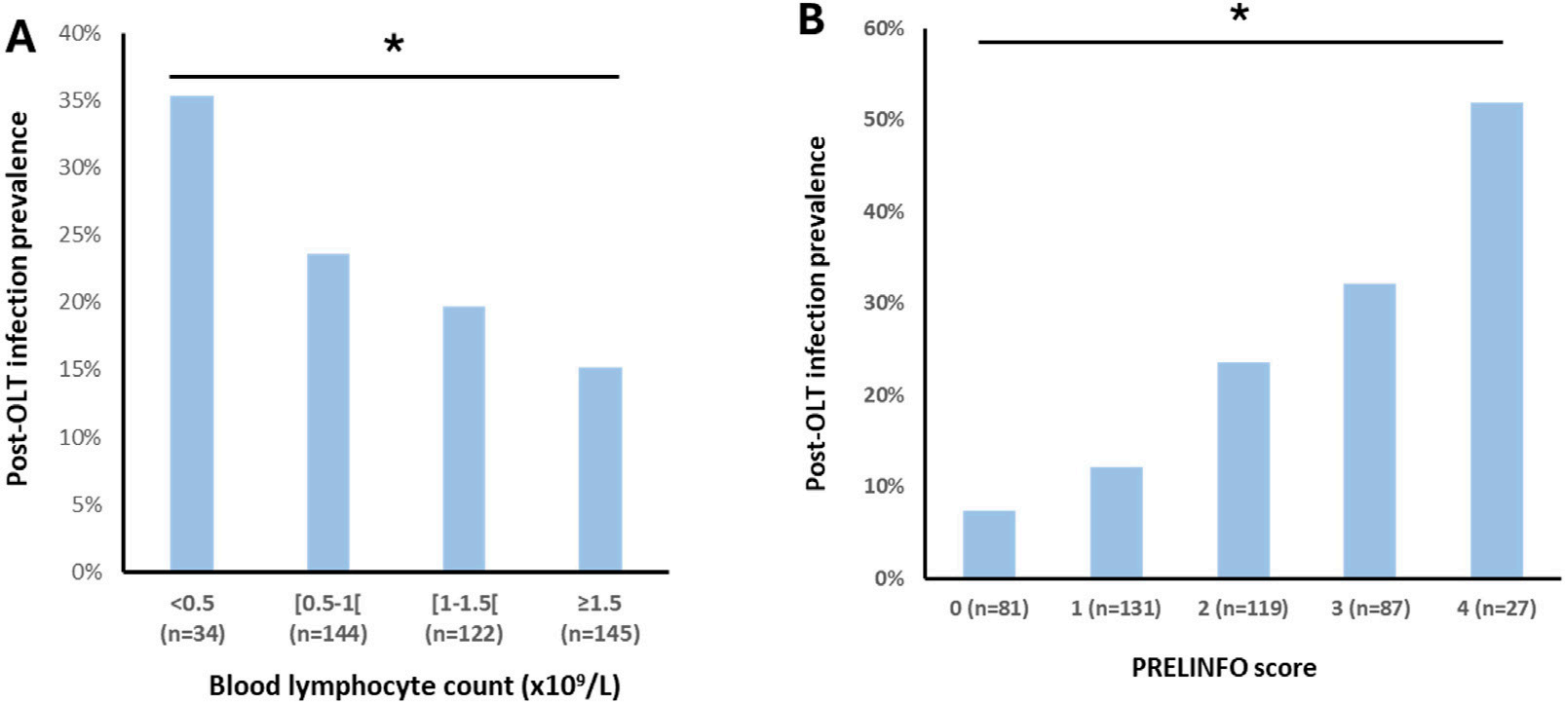
Post-OLT infection prevalence according to preoperative blood lymphocytes count (panel **(A)**) and PRELINFO score (panel **(B)**) Chi-square test used. Bold stars indicate statistically significant difference (p < 0.05).

We further analyzed the prevalence of post-OLT infections in different sub-groups according to the preoperative blood lymphocyte count: <0.5 × 10^9^/L, between 0.5 and 1.0 × 10^9^/L, between 1.0 and 1.5 × 10^9^/L and ≥1.5 × 10^9^/L. Results are displayed in Figure 2A. The lower the preoperative blood lymphocyte count, the higher the prevalence of post-OLT infections (*p* = 0.047).

### Risk Factors for Post-OLT Infections

By multivariate regression analysis with backward elimination, preoperative encephalopathy, lymphocytes ≤1.150 × 10^9^/L, intraoperative RBC transfusion >2 U and intraoperative maximum norepinephrine dose >0.5 μg.kg^−1^.min^−1^ were independent predictors of post-OLT infections (Table 2). Bootstrapping methods (2000 resampling) confirmed that they were all independent predictors of post-OLT infections. Results are displayed in Supplementary Table S3.

**TABLE 2:** Features associated with the primary endpoint by a multivariable logistic regression model.

Risk factor	OR [IC 95%]	*p*	Point
Preoperative encephalopathy	1.764 [1.047–2.974]	**0.033**	**1**
Lymphocytes≤1.15 × 10^9^/L*	1.836 [1.064–3.168]	**0.029**	**1**
RBCs transfusion>2U**	2.160 [1.242–3.755]	**0.006**	**1**
Maximum norepinephrine dose>0.5 μg.kg^−1^.min^−1^**	2.457 [1.406–4.296]	**0.002**	**1**

Multivariable logistic regression with backward elimination (exit *p* = 0.05). Results presented as OR [CI 95%] (*p*). p-value <0.05 was considered significant. p-values in bold are significant. RBC, red blood cells. * Immediate preoperative data. **Intraoperative data.

The sensitivity and specificity associated with the risk of post-OLT infection of each criterion were as follows: preoperative encephalopathy: 53% and 66%; blood lymphocyte count <1.150 × 10^9^/L: 66% and 52%; intraoperative RBC transfusion >2 U: 39% and 82%; intraoperative maximum norepinephrine dose >0.5 μg.kg^−1^.min^−1^: 72% and 51%, respectively.

### PRELINFO Score

Since the beta coefficients were roughly similar for these 4 risk factors of post-OLT infections, a score of one point was then attributed to each of them to build the PRELINFO (PRediction of EarLy INfection Following Orthotopic liver transplantation) score. The PRELINFO score ranged from 0 to 4 points. The prevalence of post-OLT infections for PRELINFO score of 0, 1, 2, 3 and 4 points was respectively 7.4%, 12.2%, 23.5%, 32.2% and 51.9% (p < 0.05) (Figure 2B). Thus, patients with a score of 0 (18%) or 1 (12%) had a low risk, while patients with a score of 3 (20%) or 4 (6%) had a medium or high risk of post-OLT infections.

We assessed the specific contribution of adding lymphopenia to the score in predicting infectious risk. Among patients who developed a postoperative infection (n = 92), 66% (n = 61) were effectively reclassified into a higher-risk category when lymphopenia was included in the score: including 24% (n = 22) in low-risk groups (0–2), and 42% (n = 39) in higher-risk groups (3–4). Conversely, among patients who did not develop a postoperative infection (n = 353), 30% were erroneously reclassified to a higher-risk category in the low-risk groups, and 18% in higher-risk groups.

### Evolution of Blood Lymphocyte Count According to the Primary Endpoint Before and After OLT

**FIGURE 3:**
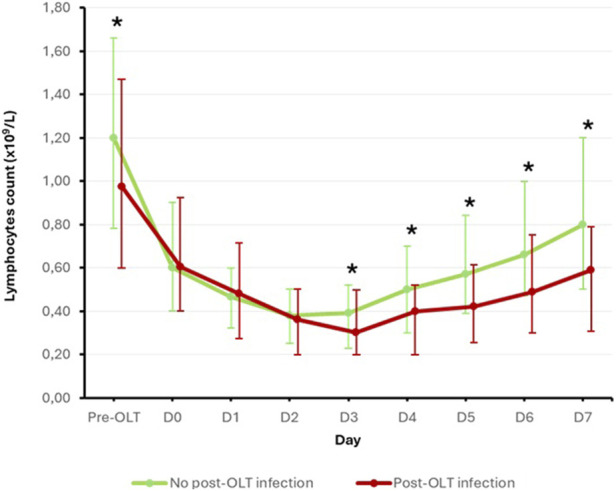
Evolution of blood lymphocyte count according to the primary endpoint Mann-Withney U test used. Results are presented as median [interquartile range]. Bold star indicates statistically significant difference (p < 0.05).

Finally, to better understand the role of preoperative lymphopenia in the occurrence of post-OLT infections, we analyzed the evolution of blood lymphocyte count before and within the first 7 days after OLT in the two subpopulations according to the occurrence of the primary endpoint. Results are displayed in Figure 3. All patients experienced a decrease in their blood lymphocyte count in the first few days post-OLT. Interestingly, while blood lymphocyte counts decreased to similar levels from D0 to D2 in both groups, patients in the post-OLT infection group showed a statistically slower resurgence in blood lymphocyte counts from D3 onwards than patients in the no post-OLT infection group.

### Subgroup Analysis

In our cohort, 312 patients (70%) were transplanted for decompensated liver disease and 133 (30%) for HCC with compensated cirrhosis. HCC was a protective factor for the occurrence of post-OLT infection (OR = 0.57 [0.361–0.922], *p* = 0.021). In the HCC-compensated subgroup, 19 (14%) patients developed a post-OLT infection, and preoperative lymphocyte count was not significantly associated with infection risk (*p* = 0.870). We conducted a secondary analysis in the decompensated cirrhosis subgroup (n = 312) which included 73 (23%) patients who developed a post-OLT infection. The results confirmed the data obtained on the main population and are presented in Supplementary Tables S4, S5. As in the main cohort, the PRELINFO score was associated with the occurrence of post-LT infection (*p* < 0.001).

## Discussion

In our study, 21% of patients presented at least one bacterial infectious complication during the hospitalization in intensive care following OLT for cirrhosis. These infections, mainly represented by bacteremia, pneumonia and surgical site infection, occurred in median 5 days after the transplantation. In our cohort, we have highlighted that the lower the preoperative blood lymphocyte count, the higher the prevalence of infections. In multivariate analysis, preoperative blood lymphocyte count ≤1.150 × 10^9^/L was found to be an independent risk factor for early post-OLT infections as was preoperative MELD ≥25, and intraoperative RBC transfusion >2 U during the liver transplantation. These parameters were integrated into a predictive score for early bacterial infections following liver transplantation: the PRELINFO score. The higher the score, the greater the risk of early post-OLT infections. Finally, the kinetics of lymphocyte count during the first seven days after OLT differed between patients who would develop a postoperative infection and those who would not. The preoperative lymphocyte count was lower, and while all patients were similarly lymphopenic in the early postoperative days, the recovery from lymphopenia was slower in the post-OLT infection group.

Infections are a major cause of morbidity and mortality after OLT [3, 12, 26, 27]. The incidence of infections reported in the literature can reach 80% in the year following the transplantation [26]. In the vast majority of cases (around 70% of cases), these are bacterial infections [12, 26], and the early postoperative period seems to be particularly at risk [1, 28]. Indeed, 20%–40% of patients would develop a bacterial infection within the first month following the OLT [3, 12]. In our study, we found similar results to the literature in terms of infected sites (bacteremia, pneumonia and surgical site infections) [3, 12, 28] but the infection rate was in the low range of what is described in the literature (21%). This can be explained on the one hand by the fact that only infections occurring during ICU hospitalization (median length of stay 8 days [6–12]) have been collected, and on the other hand by the study population which was relatively selected (inclusion of cirrhotic patients exclusively, exclusion of re-transplantations, multiple transplantations, patients with immediate preoperative infection or suspicion of intraoperative infection especially). Possibly for the same reasons, we observed fewer septic shocks than described by Laici et al. in 2018 [3] who found that post-OLT infections were complicated by a septic shock in almost a quarter of cases and were responsible for almost half of the deaths occurring at day 90.

There were several reasons for analyzing the relationship between lymphopenia and post-OLT infections. First, the risk of bacterial infection after solid organ transplantation seems to increase with the degree of immunosuppression [29]. The latter depends not only on the immunosuppressive treatments introduced after the transplantation but also on the pre-existing level of immunosuppression specific to each patient [1, 12]. Since a few years, alterations in the immune response in cirrhotic patients have been described under the term “Cirrhosis-Associated Immune Dysfunction,” for which lymphopenia is thought to have an important role [14, 15, 30]. Moreover, preoperative lymphocyte count was associated with the occurrence of infection in the subgroup of patients with decompensated cirrhosis and was not in the subgroup of patients transplanted for HCC with compensated cirrhosis. This is consistent with the fact that CAID, and hence lymphopenia, is more pronounced in patients with advanced cirrhosis. Thus, the absolute lymphocyte count could be a simple and accessible marker to easily assess the basal immunosuppression state of cirrhotic patients before OLT. Recently, lymphopenia at the time of the liver transplantation has been associated with short-term mortality [31]. In this study, the authors found that patients with very low preoperative lymphocyte count (<500/µL) had a higher risk of mortality, particularly sepsis-related mortality, and of bacteremia within 180 days post-OLT. However, this study was not designed to assess the relationship between preoperative blood lymphocyte count and early postoperative infections. Our results provide new data by considering all bacterial sepsis and focusing on the early postoperative period, known to be particularly at risk [1, 28]. Moreover, we demonstrate an effect of lymphopenia on the occurrence of infections from a higher lymphocyte threshold (<1.15 × 10^9^/L). Another study showed that patients who developed infection after OLT had a higher neutrophil-to-lymphocyte ratio the day before the sepsis than those who did not, suggesting that a low postoperative lymphocyte count is associated with the risk of infection after OLT [32]. However, this study failed to show an effect between preoperative neutrophil-to-lymphocyte ratio or preoperative lymphocyte count and postoperative infections. In our study, we analyzed the preoperative neutrophil-to-lymphocyte ratio which was less accurate than lymphocyte count in predicting early postoperative infections (data not shown). Finally, Riff et al. found similar results with regard to the kinetics of post-OLT lymphocyte count in patients with cirrhosis [33]. Thus, to the best of our knowledge, our study is the first to show a clear association between preoperative lymphocyte count and the occurrence of early post-OLT bacterial infections in patients with cirrhosis.

Secondly, treatments limiting immune rejection have a major role in the postoperative immunosuppression state [29, 34]. The main immunosuppressive treatments used after OLT (glucocorticoids, tacrolimus and mycophenolate mofetil) all cause qualitative or quantitative lymphocyte alterations, thus worsening the potential pre-existing lymphopenia. Although the risk of post-OLT rejection is becoming low, in particular by the improvement in immunosuppressive treatments, the balance between risk of infection and risk of transplant rejection remains challenging [35]. A recent national survey, assessing perioperative management practices, found that 30% of OLT centers modified the immunosuppressive regimen (mainly by reducing tacrolimus or corticosteroid doses) in case of postoperative suspected sepsis [36]. In the future, studies will be needed to determine the value of preoperative blood lymphocyte count to individualize the immunosuppressive regimen in the immediate postoperative period based on the assessment of post-OLT infection risk.

Among the other risk factors for post-OLT infections described in the literature, the MELD score is inconsistently found. Some studies did not find an association between MELD and the occurrence of surgical site infection [37], pneumonia or bacteremia after OLT [38]. Conversely, a study published in 2013 by Avkan-Oguz et al. found an association between a MELD score >20 and the onset of bacterial infections within 30 days after the OLT, whatever the infected sites [5]. In our study, in multivariate analysis, the history of encephalopathy was a more accurate factor than MELD in reflecting the impact of cirrhosis severity on the risk of post-OLT infection. Intraoperative RBC transfusion has also been studied by other teams and is frequently described as a predictor of post-OLT infections [5, 6, 39, 40]. However, while the immunosuppressive role of transfusion is recognized [41], the significant thresholds in terms of transfusion volume vary between studies [5, 6]. While Avkan-Oguz et al. [5] considered a transfusion threshold greater than 6 U as a risk factor for post-OLT infections, the threshold we used in our study (>2U) was based on the evaluation of the Youden index and can be explained by a very low median of intraoperative transfusion in our cohort (1 U [0–2]). Rarely described in the literature in this way, we also show in our study a link between intraoperative severity represented by the maximum norepinephrine dose and the risk of early post-OLT infection. Other risk factors have been described in the literature such as undernutrition, renal-replacement therapy, the need for retransplantation, history of COPD or even a Roux-en-Y anastomosis biliary reconstruction [3, 5, 37]. Interestingly, the type of biliary reconstruction was not identified as a risk factor in our work, but this is most likely linked to the very high predominance of duct-to-duct reconstruction in our cohort (92%).

We propose a simple, pragmatic score for bedside use in the immediate post-OLT period to assess the risk of early infection. The preoperative blood lymphocyte count and the PRELINFO score could be used to adapt the immunosuppression regimen and indicate closer monitoring of the development of bacterial infection or even pursue more prolonged antibiotic prophylaxis in patients most at risk.

Our study has several limitations. First, it is a monocentric study. Although many characteristics of our population are consistent with what is reported in the literature, the results we present need to be validated in an external cohort. To be used in everyday practice, the PRELINFO score would need to be validated in another prospective, multicenter study. In order to limit this bias, we used a bootstrap analysis providing an internal validation of the multivariable logistic regression model. Second, although the database was completed prospectively, some specific data of our work have been collected retrospectively potentially biasing the results. Furthermore, we cannot rule out the possibility that certain data that we were unable to collect may have affected lymphocyte levels (e.g., certain patient-specific treatments) or the risk of infection (e.g., cumulative dose of tacrolimus or mycophenolate mofetil, hypogammaglobulinemia). Finally, the study period is relatively long. It is possible that medical and surgical practices have evolved over time, thus influencing the results we have observed.

In conclusion, early bacterial infections after OLT for cirrhosis are a relatively frequent phenomenon and represent a real challenge in terms of morbidity and mortality in the early post-operative period. We highlight several known risk factors and the role of preoperative lymphopenia in the occurrence of these infections. These results suggest that preoperative blood lymphocyte count should be incorporated into the assessment of the risk of post-OLT bacterial infections, and that further studies should be carried out to clarify its use in defining the immunosuppression regimen in the early postoperative period.

## Data Availability

The data analyzed in this study is subject to the following licenses/restrictions: The datasets used and/or analysed during the current study are available from the corresponding author on reasonable request. Requests to access these datasets should be directed to MG mikhael.giabicani@aphp.fr.

## Abbreviations

OLT, orthotopic liver transplantation; ICU, intensive care unit; MELD, Model for End-Stage Liver Disease; ACLF, Acute-on-Chronic Liver Failure; CAID, cirrhosis associated immune dysfunction; RBC, red blood cell; BPSS, blind protected bronchial sampling; BAL, bronchoalveolar lavage; CFU, colony-forming units; SSI, surgical-site infections; UTI, urinary tract infections; ESBL-E, Extended-Spectrum β-Lactamase-producing *Enterobacteriaceae*; PRELINFO, PRediction of EarLy INfection Following Orthotopic liver transplantation; HCC, hepatocellular carcinoma.

## References

[1] Timsit JF Sonneville R Kalil AC Bassetti M Ferrer R Jaber S Diagnostic and Therapeutic Approach to Infectious Diseases in Solid Organ Transplant Recipients. Intensive Care Med (2019) 45(5):573–91. 10.1007/s00134-019-05597-y 30911807 PMC7079836

[2] Jerome E Cavazza A Menon K McPhail MJ. Systematic Review and Meta-Analysis of the Diagnostic Accuracy of Procalcitonin for Post-Operative Sepsis/Infection in Liver Transplantation. Transpl Immunol (2022) 74:101675. 10.1016/j.trim.2022.101675 35878844

[3] Laici C Gamberini L Bardi T Siniscalchi A Reggiani MLB Faenza S. Early Infections in the Intensive Care Unit after Liver Transplantation-Etiology and Risk Factors: A Single-Center Experience. Transpl Infect Dis (2018) 20(2):e12834. 10.1111/tid.12834 29359867

[4] Elkholy S Mansour DA El-Hamid S Al-Jarhi UM El-Nahaas SM Mogawer S. Risk Index for Early Infections Following Living Donor Liver Transplantation. Arch Med Sci (2019) 15(3):656–65. 10.5114/aoms.2019.84736 31110531 PMC6524199

[5] Avkan-Oguz V Ozkardesler S Unek T Ozbilgin M Akan M Firuzan E Risk Factors for Early Bacterial Infections in Liver Transplantation. Transpl Proc (2013) 45(3):993–7. 10.1016/j.transproceed.2013.02.067 23622606

[6] Vera A Contreras F Guevara F. Incidence and Risk Factors for Infections After Liver Transplant: Single-Center Experience at the University Hospital Fundación Santa Fe de Bogotá, Colombia. Transpl Infect Dis (2011) 13(6):608–15. 10.1111/j.1399-3062.2011.00640.x 21794041

[7] Kim SI. Bacterial Infection After Liver Transplantation. World J Gastroenterol (2014) 20(20):6211–20. 10.3748/wjg.v20.i20.6211 24876741 PMC4033458

[8] Hannon VN Tinguely P McKenna GJ Brustia R Kaldas FM Scatton O New ERAS in Liver Transplantation - Past, Present and Next Steps. Clin Transpl (2022) 36:e14625. 10.1111/ctr.14625 35238415

[9] Taner CB Willingham DL Bulatao IG Shine TS Peiris P Torp KD Is a Mandatory Intensive Care Unit Stay Needed after Liver Transplantation? Feasibility of Fast-Tracking to the Surgical Ward after Liver Transplantation. Liver Transpl (2012) 18(3):361–9. 10.1002/lt.22459 22140001

[10] Bulatao IG Heckman MG Rawal B Aniskevich S Shine TS Keaveny AP Avoiding Stay in the Intensive Care Unit After Liver Transplantation: A Score to Assign Location of Care. Am J Transpl (2014) 14(9):2088–96. 10.1111/ajt.12796 25088768

[11] Ichai P. Infection after Liver Transplantation. La lettre de l’infectiologue Tome (2012).

[12] Pedersen M Seetharam A. Infections after Orthotopic Liver Transplantation. J Clin Exp Hepatol (2014) 4(4):347–60. 10.1016/j.jceh.2014.07.004 25755581 PMC4298628

[13] Adam R Karam V Cailliez V O Grady JG Mirza D Cherqui D 2018 Annual Report of the European Liver Transplant Registry (ELTR) - 50-Year Evolution of Liver Transplantation. Transpl Int (2018) 31(12):1293–317. 10.1111/tri.13358 30259574

[14] Albillos A Lario M Álvarez-Mon M. Cirrhosis-Associated Immune Dysfunction: Distinctive Features and Clinical Relevance. J Hepatol (2014) 61(6):1385–96. 10.1016/j.jhep.2014.08.010 25135860

[15] Albillos A Martin-Mateos R Van der Merwe S Wiest R Jalan R Álvarez-Mon M. Cirrhosis-Associated Immune Dysfunction. Nat Rev Gastroenterol Hepatol (2022) 19(2):112–34. 10.1038/s41575-021-00520-7 34703031

[16] McGovern BH Golan Y Lopez M Pratt D Lawton A Moore G The Impact of Cirrhosis on CD4+ T Cell Counts in HIV-Seronegative Patients. Clin Infect Dis (2007) 44(3):431–7. 10.1086/509580 17205454

[17] Weiss E de la Grange P Defaye M Lozano JJ Aguilar F Hegde P Characterization of Blood Immune Cells in Patients with Decompensated Cirrhosis Including ACLF. Front Immunol (2020) 11:619039. 10.3389/fimmu.2020.619039 33613548 PMC7893087

[18] Clària J Arroyo V Moreau R. Roles of Systemic Inflammatory and Metabolic Responses in the Pathophysiology of Acute-On-Chronic Liver Failure. JHEP Rep (2023) 5(9):100807. 10.1016/j.jhepr.2023.100807 37600957 PMC10432809

[19] Olthoff KM Kulik L Samstein B Kaminski M Abecassis M Emond J Validation of a Current Definition of Early Allograft Dysfunction in Liver Transplant Recipients and Analysis of Risk Factors. Liver Transpl (2010) 16(8):943–9. 10.1002/lt.22091 20677285

[20] Moreau R Jalan R Gines P Pavesi M Angeli P Cordoba J Acute-on-Chronic Liver Failure Is a Distinct Syndrome that Develops in Patients with Acute Decompensation of Cirrhosis. Gastroenterology (2013) 144(7):1426–37.e14379. 10.1053/j.gastro.2013.02.042 23474284

[21] Logre E Bert F Khoy-Ear L Janny S Giabicani M Grigoresco B Risk Factors and Impact of Perioperative Prophylaxis on the Risk of Extended-Spectrum β-Lactamase-Producing Enterobacteriaceae-Related Infection Among Carriers Following Liver Transplantation. Transplantation (2021) 105(2):338–45. 10.1097/TP.0000000000003231 32217945

[22] American Thoracic Society, Infectious Diseases Society of AmericaInfectious Diseases Society of America. Guidelines for the Management of Adults with Hospital-Acquired, Ventilator-Associated, and Healthcare-Associated Pneumonia. Am J Respir Crit Care Med (2005) 171(4):388–416. 10.1164/rccm.200405-644ST 15699079

[23] Anonymous Surgical Site Infection (2021);39.

[24] Hooton TM Bradley SF Cardenas DD Colgan R Geerlings SE Rice JC Diagnosis, Prevention, and Treatment of Catheter-Associated Urinary Tract Infection in Adults: 2009 International Clinical Practice Guidelines from the Infectious Diseases Society of America. Clin Infect Dis (2010) 50(5):625–63. 10.1086/650482 20175247

[25] Bert F Larroque B Paugam-Burtz C Dondero F Durand F Marcon E Pretransplant Fecal Carriage of Extended-Spectrum β-lactamase-producing Enterobacteriaceae and Infection after Liver Transplant, France. Emerg Infect Dis (2012) 18(6):908–16. 10.3201/eid1806.110139 22607885 PMC3358139

[26] Romero FA Razonable RR. Infections in Liver Transplant Recipients. World J Hepatol (2011) 3(4):83–92. 10.4254/wjh.v3.i4.83 21603030 PMC3098392

[27] Reid GE Grim SA Sankary H Benedetti E Oberholzer J Clark NM. Early Intra-Abdominal Infections Associated With Orthotopic Liver Transplantation. Transplantation (2009) 87(11):1706–11. 10.1097/TP.0b013e3181a60338 19502964

[28] Hernandez MDP Martin P Simkins J. Infectious Complications after Liver Transplantation. Gastroenterol Hepatol (N Y) (2015) 11(11):741–53. 27134589 PMC4849501

[29] Fishman JA. Infection in Organ Transplantation. Am J Transpl (2017) 17(4):856–79. 10.1111/ajt.14208 28117944

[30] Romo EM Muñoz-Robles JA Castillo-Rama M Meneu JC Moreno-Elola A Pérez-Saborido B Peripheral Blood Lymphocyte Populations in End-Stage Liver Diseases. J Clin Gastroenterol (2007) 41(7):713–21. 10.1097/01.mcg.0000248000.42581.35 17667057

[31] Kitajima T Rajendran L Lisznyai E Lu M Shamaa T Ivanics T Lymphopenia at the Time of Transplant Is Associated with Short-Term Mortality after Deceased Donor Liver Transplantation. Am J Transpl (2023) 23(2):248–56. 10.1016/j.ajt.2022.11.004 36804132

[32] Sarin S Pamecha V Sinha PK Patil N Mahapatra N. Neutrophil Lymphocyte Ratio Can Preempt Development of Sepsis after Adult Living Donor Liver Transplantation. J Clin Exp Hepatol (2022) 12(4):1142–9. 10.1016/j.jceh.2021.11.008 35814504 PMC9257924

[33] Riff A Haem RM Delignette MC Gossez M Coudereau R Pantel S Assessment of Neutrophil Subsets and Immune Checkpoint Inhibitor Expressions on T Lymphocytes in Liver Transplantation: A Preliminary Study beyond the Neutrophil-Lymphocyte Ratio. Front Physiol (2023) 14:1095723. 10.3389/fphys.2023.1095723 37064910 PMC10097891

[34] Duncan MD Wilkes DS. Transplant-Related Immunosuppression: A Review of Immunosuppression and Pulmonary Infections. Proc Am Thorac Soc (2005) 2(5):449–55. 10.1513/pats.200507-073JS 16322599 PMC2713333

[35] Weiss E Restoux A Paugam-Burtz C. Anesthésie-réanimation en transplantation hépatique. Le Praticien en Anesthésie Réanimation (2019) 23(2):56–64. 10.1016/j.pratan.2019.02.008

[36] Devauchelle P Bignon A Breteau I Defaye M Degravi L Depres C Perioperative Management During Liver Transplantation: A National Survey from the French Special Interest Group in “Liver Anesthesiology and Intensive Care”. Transplantation (2025) 109:671–80. in press. 10.1097/TP.0000000000005264 40071909

[37] Freire MP Soares Oshiro ICV Bonazzi PR Guimarães T Ramos Figueira ER Bacchella T Surgical Site Infections in Liver Transplant Recipients in the Model for End-Stage Liver Disease Era: An Analysis of the Epidemiology, Risk Factors, and Outcomes. Liver Transpl (2013) 19(9):1011–9. 10.1002/lt.23682 23744748

[38] Juntermanns B Manka P Hoyer DP Kaiser GM Radunz S Pracht W Infectious Complications in the Era of MELD. Ann Transpl (2015) 20:297–302. 10.12659/AOT.893122 26017072

[39] Benson AB Burton JR Austin GL Biggins SW Zimmerman MA Kam I Differential Effects of Plasma and Red Blood Cell Transfusions on Acute Lung Injury and Infection Risk Following Liver Transplantation. Liver Transpl (2011) 17(2):149–58. 10.1002/lt.22212 21280188 PMC3399914

[40] Nierenberg NE Poutsiaka DD Chow JK Cooper J Price LL Freeman RB Pretransplant Lymphopenia Is a Novel Prognostic Factor in Cytomegalovirus and Noncytomegalovirus Invasive Infections after Liver Transplantation. Liver Transpl (2014) 20(12):1497–507. 10.1002/lt.23991 25205044 PMC4451230

[41] Remy KE Hall MW Cholette J Juffermans NP Nicol K Doctor A Mechanisms of Red Blood Cell Transfusion-Related Immunomodulation. Transfusion (2018) 58(3):804–15. 10.1111/trf.14488 29383722 PMC6592041

