# Supplementary material for: Role of Lymphopenia in Early prediction of Infection Following Orthotopic Liver Transplantation in Cirrhotic Patients
Source: Transpl Int. 2025 May 12;38:14372. doi: 10.3389/ti.2025.14372 (PMC12105490; doi:10.3389/ti.2025.14372)
Supplement: Supplementary file 1 [file DataSheet1.docx]

**Capsule sentence summary:**

Preoperative lymphocyte count≤1.150x10^9^/L was identified as an independent risk factor for early bacterial infection following liver transplantation and was integrated, with other risk factors (encephalopathy, intraoperative RBC transfusion>2, and norepinephrine>0.5µg.kg^-1^.min^-1^) into the PRELINFO score which should be used to assess the risk of infection.

**Graphical abstract**

**
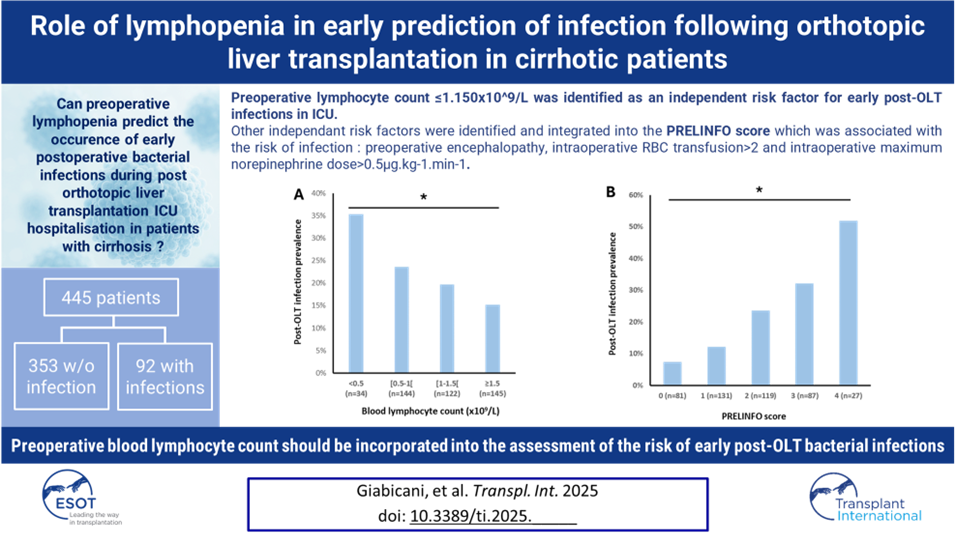
**

**Table S1.**

|  | **All (n=445)** | **Post-LT infection (n=92)** | **Absence of post-LT infection (n=353)** | ***p*** |
| --- | --- | --- | --- | --- |
| Donor’s age (y) | 60 [44-77] | 62 [45-78] | 59 [43-77] | 0.779 |
| Brain dead donor, n (%) | 391 (88) | 84 (91) | 307 (87) | 0.400 |
| Donor’s BMI (kg.m^-2^) | 25 [22-28] | 26 [23-28] | 25 [22-28] | 0.276 |
| Graft cold ischemia time (min) | 425 [357-534] | 432 [374-540] | 420 [351-535] | 0.570 |
| Graft warm ischemia time (min) | 45 [38-53] | 45 [36-55] | 45 [39-52] | 0.904 |

Mann-Whitney U test used for continuous variables. Chi-square test used for categorical variables. Results are expressed as number (percentage) or median [interquartile range]. p-value<0.05 was considered significant. p-values in bold are significant. BMI, body mass index.

**Table S2.** Post-OLT infections related morbidity and mortality

|  | **All (n=445)** | **Post-LT infection (n=92)** | **Absence of post-LT infection (n=353)** | ***p*** |
| --- | --- | --- | --- | --- |
| Duration under mechanical ventilation (d) | 0.24 [0.1-0.5] | 0.4 [0.16-2.0] | 0.2 [0.08-0.48] | **<0.001** |
| Vasopressor infusion duration (d) | 0.25 [0.8-1.0] | 0.53 [0.2-1.08] | 0.2 [0.04-0.75] | **<0.001** |
| Renal replacement therapy, n (%) | 34 (8) | 20 (22) | 14 (4) | **<0.001** |
| ICU length of stay (d) | 8 [6-12] | 16 [11-23] | 7 [6-10] | **<0.001** |
| 30-day mortality, n (%) | 11 (2) | 8 (9) | 3 (1) | **<0.001** |
| 90-day mortality, n (%) | 15 (3) | 10 (11) | 5 (1) | **<0.001** |

Mann-Whitney U test used for continuous variables. Chi-square test used for categorical variables. Results are expressed as number (percentage) or median [interquartile range]. p-value<0.05 was considered significant. p-values in bold are significant. ICU, Intensive Care Unit.

**Table S3. Bootstrap analysis**

|  | β coefficient | IC [95%] | *p* |
| --- | --- | --- | --- |
| Preoperative encephalopathy | 0.568 | [0.033-1.105] | **0.032** |
| Lymphocytes≤1.15x10^9^/L* | 0.608 | [0.046-1.220] | **0.036** |
| RBCs transfusion>2U** | 0.770 | [0.221-1.334] | **0.005** |
| Maximum norepinephrine dose>0.5 µg.kg^-1^.min^-1^** | 0.899 | [0.369-1.525] | **0.001** |

Bootstrap analysis with 2000 resampling in multivariable logistic regression with backward elimination (exit p = 0.05). Results presented as β coefficient [CI 95%] (*p*). RBC, red blood cells. * Immediate preoperative data. **Intraoperative data.

**Table S4. Patients’ main characteristics and univariate analysis in the decompensated cirrhosis subgroup**

|  | **All (n=312)** | **Post-LT infection (n=73)** | **Absence of post-LT infection (n=239)** | ***p*** |
| --- | --- | --- | --- | --- |
| **Baseline characteristics** | | | | |
| Age (years) | 57 [50-63] | 57 [50-63] | 57 [51-62] | 0.819 |
| Male sex, n (%) | 229 (73) | 53 (73) | 176 (74) | 0.861 |
| BMI (kg.m^-2^) | 26 [23-30] | 26 [24-30] | 26 [23-30] | 0.857 |
| Malnutrition, n (%) | 144 (46) | 40 (55) | 104 (44) | 0.091 |
| Diabetes, n (%) | 80 (26) | 20 (27) | 60 (25) | 0.618 |
| HIV coinfection, n (%) | 4 (1) | 2 (3) | 2 (1) | 0.208 |
| COPD, n (%) | 6 (2) | 1 (1) | 5 (2) | 0.704 |
| Cause of cirrhosis, n (%)   - Excessive alcohol consumption - Metabolic syndrome - HCV infection - HBV infection - Auto-immune hepatitis - Cholestatic liver disease | 187 (60)  84 (27)  55 (17)  31 (10)  18 (6)  15 (5) | 43 (59)  20 (27)  13 (18)  4 (5)  5 (7)  3 (4) | 144 (60)  64 (27)  42 (18)  27 (11)  13 (5)  12 (5) | 0.837  0.917  0.963  0.146  0.651  0.750 |
| HCC, n (%) | 90 (29) | 17 (23) | 73 (31) | 0.231 |
| Preoperative ascites, n (%) | 140 (45) | 33 (45) | 107 (45) | 0.948 |
| Preoperative encephalopathy, n (%) | 169 (54) | 49 (67) | 120 (50) | **0.011** |
| History of SBP, n (%) | 76 (24) | 22 (30) | 54 (23) | 0.189 |
| Severity of cirrhosis   - MELD - MELD≥ 25, n (%) - ACLF, n (%) | 17 [12-22]  54 (17)  34 (11) | 18 [14-26]  20 (27)  14 (19) | 17 [11-21]  34 (14)  20 (8) | **0.015**  **0.010**  **0.009** |
| **Pre-LT blood count** | | | | |
| Hemoglobin (g/L)  Hemoglobin<11 g/L, n (%) | 112 [96-124]  140 (45) | 104 [90-119]  43 (59) | 113 [96-126]  97 (239) | **0.020**  **0.004** |
| Platelets (x10^9^/L) | 86 [63-121] | 80 [59-126] | 89 [66-120] | 0.351 |
| Leucocytes (x10^9^/L) | 5.00 [3.90-6.80] | 5.10 [3.60-7.10] | 5.00 [4.00-6.80] | 0.930 |
| Neutrophils (x10^9^/L) | 3.08 [2.25-4.20] | 3.19 [2.45-4.66] | 3.00 [2.20-4.20] | 0.237 |
| Lymphocytes (x10^9^/L) | 1.00 [0.65-1.50] | 0.80 [0.55-1.23] | 1.03 [0.70-1.50] | **0.009** |
| Lymphocytes≤1.15x10^9^/L, n (%) | 189 (61) | 55 (75) | 134 (56) | **0.003** |
| Monocytes (x10^9^/L) | 0.60 [0.40-0.84] | 0.59 [0.36-0.88] | 0.60 [0.40-0.80] | 0.908 |
| **Intraoperative characteristics** | | | | |
| Surgery duration (min) | 310 [275-359] | 323 [270-378] | 310 [276-351] | 0.385 |
| Cold ischemia time (min) | 425 [356-533] | 432 [374-532] | 422 [347-535] | 0.717 |
| Warm ischemia time (min) | 45 [38-51] | 45 [36-51] | 45 [38-51] | 0.727 |
| Blood loss (mL)  Blood loss≥750 | 1000 [600-1600]  197 (63) | 1000 [800-2000]  53 (73) | 1000 [550-1550]  144 (60) | **0.013**  **0.015** |
| RBC transfusion, n (%) | 196 (63) | 56 (77) | 140 (59) | **0.005** |
| Number of RBCs units transfused (U) | 2 [0-3] | 2 [1-4] | 1 [0-2] | **<0.001** |
| RBCs transfusion>2U (%) | 94 (30) | 35 (48) | 59 (25) | **<0.001** |
| Reperfusion syndrome, n (%) | 164 (53) | 39 (53) | 125 (52) | 0.866 |
| Maximum norepinephrine dose (µg.kg^-1^.min^-1^)  Maximum norepinephrine dose>0.5 µg.kg^-1^.min^-1^, n (%) | 0.60 [0.37-0.98]  193 (62) | 0.72 [0.52-1.20]  55 (75) | 0.57 [0.32-0.93]  138 (58) | **0.003**  **0.007** |
| Biliary reconstruction, n (%)   - Duct-to-duct - Roux-en-Y anastomosis | 284 (91)  6 (2) | 67 (92)  1 (1) | 217 (91)  5 (2) | 0.692  0.692 |

Mann-Whitney U test used for continuous variables. Chi-square test used for categorical variables. Results are expressed as number (percentage) or median [interquartile range]. p-value<0.05 was considered significant. p-values in bold are significant. BMI, body mass index; HIV, Human Immunodeficiency Virus; COPD, chronic obstructive pulmonary disease; HCV, hepatitis C virus; HBV, hepatitis B virus; HCC, hepatocellular carcinoma; SBP, spontaneous bacterial peritonitis; MELD: Model for End-stage Liver Disease; ACLF, Acute on Chronic Liver Failure; RBC, red blood cells.

**Table S5.** **Features associated with the primary endpoint by a multivariable logistic regression model in the decompensated cirrhosis subgroup**

|  | **OR [IC 95%]** | ***p*** | ***Point*** |
| --- | --- | --- | --- |
| Preoperative encephalopathy | 1.995 [1.088-3.659] | **0.026** | **1** |
| Lymphocytes≤1.15x10^9^/L* | 2.492 [1.277-4.863] | **0.007** | **1** |
| RBCs transfusion>2U** | 2.239 [1.239-4.045] | **0.008** | **1** |
| Maximum norepinephrine dose>0.5 µg.kg^-1^.min^-1^** | 2.136 [1.116-4.090] | **0.022** | **1** |

Multivariable logistic regression with backward elimination (exit *p* = 0.05). Results presented as OR [CI 95%] (*p*). p-value<0.05 was considered significant. p-values in bold are significant. RBC, red blood cells. * Immediate preoperative data. **Intraoperative data.
